# A Natural Peptide from the American Cockroach Suppresses Feline Infectious Peritonitis Virus

**DOI:** 10.3390/biology15151324

**Published:** 2026-08-06

**Authors:** Tengyu Zhu, Jinfeng Hou, Tong Jing, Yibo Wu, Rui Meng, Ruobing Liu, Kun Yuan, Sunchengai Cao, Xinyan Yang, Jiayi Wang, Ziyin Wang, Zhengyan Zhu, Yaogui Sun, Lin Jin

**Affiliations:** Shanxi Key Laboratory for Modernization of TCVM, College of Veterinary Medicine, Shanxi Agricultural University, Taiyuan 030031, China; zhutengyu@sxau.edu.cn (T.Z.); 202430895@stu.sxau.edu.cn (J.H.); 20222017319@stu.sxau.edu.cn (T.J.); 202420430@stu.sxau.edu.cn (Y.W.); 202430919@stu.sxau.edu.cn (R.M.); 202420404@stu.sxau.edu.cn (R.L.); 20233841@stu.sxau.edu.cn (K.Y.); 20232017024@stu.sxau.edu.cn (S.C.); 20232017004@stu.sxau.edu.cn (X.Y.); 20241312032@stu.sxau.edu.cn (J.W.); 20252018411@stu.sxau.edu.cn (Z.W.); 20252018518@stu.sxau.edu.cn (Z.Z.)

**Keywords:** feline infectious peritonitis virus, periplaneta americana, ISG15, antiviral

## Abstract

Feline infectious peritonitis virus (FIPV) causes a fatal and immune-mediated disease in cats with limited treatment options. Natural products offer promising antiviral candidates, but animal-derived peptides remain underexplored. Here, we identified PI-8, a Periplaneta americana (*P. americana*)-derived peptide with validated binding to ISG15, and evaluated its anti-FIPV effect and mechanism. PI-8 showed low cytotoxicity (CC_50_ > 40 μM) and inhibited FIPV replication with an IC_50_ of 12.9 μM, acting primarily at the post-entry stage. PI-8 significantly upregulated IFNA1, IFNB, ISG15, and MX1 in infected CRFK cells. In FIPV-infected cats, PI-8 reduced RNA levels in blood, saliva, and anal swabs, and lowered tissue viral burden in liver, spleen, lung, kidney, and intestine. It also alleviated gross and histopathological lesions and decreased inflammatory cytokine expression. These findings suggest that PI-8 is an *P. americana*-derived peptide with dual anti-FIPV and immunomodulatory activity associated with an IFN-ISG15-related antiviral response. PI-8 represents a potential host-directed therapeutic candidate for FIPV infection.

## Simple Summary

In this study, a novel ISG15-targeting peptide, PI-8, derived from Periplaneta americana, was identified, and its anti-feline infectious peritonitis virus (FIPV) efficacy was validated through both in vitro and in vivo experiments. PI-8 exhibited low cytotoxicity and characteristically suppressed FIPV replication at the post-entry stage. Concurrently, it markedly upregulated the expression of critical interferons and antiviral interferon-stimulated genes (ISGs) in FIPV-infected Crandell–Rees feline kidney (CRFK) cells. In FIPV-infected cats, administration of PI-8 diminished viral loads across various physiological specimens and visceral organs, alleviated histopathological lesions, and suppressed inflammatory responses by modulating ISG15-mediated immune signaling pathways. Collectively, these findings demonstrate that PI-8 is a dual-functional natural peptide possessing potent antiviral and immunomodulatory properties, establishing it as a promising host-directed therapeutic candidate for the treatment of FIPV infection.

## 1. Introduction

Feline infectious peritonitis (FIP) is a fatal, immune-mediated disease in cats caused by feline infectious peritonitis virus (FIPV), a virulent biotype of feline coronavirus (FCoV) that preferentially infects macrophages and triggers systemic inflammation and immune dysregulation [1]. FIPV is a positive single-stranded RNA virus and associated with enhanced replication in monocytes and macrophages, systemic dissemination, immune-mediated vasculities, serositis and pyogranulomatous inflammation [2,3,4]. FIP may present as effusive, non-effusive or in mixed forms, and its clinical progression is driven not only by viral replication but also by dysregulated host inflammatory and immune responses [2,4,5]. Although nucleoside analogs such as GS-441524 and remdesivir, as well as the 3C-like protease inhibitor GC376, have greatly changed the therapeutic landscape of FIP, challenges remain regarding drug accessibility, treatment monitoring, relapse, tissue penetration and the need for complementary host-directed strategies [6]. Therefore, the development of novel drugs capable of effectively inhibiting FIPV infection is a matter of urgent necessity.

Innate antiviral immunity is the first line of host defense against viral infection and is largely coordinated by type I interferons and interferon-stimulated genes (ISGs) [7,8]. Coronaviruses encode multiple immune-evasion proteins that antagonize interferon induction and downstream signaling [9]. In FIPV, the viral 3C-like protease Nsp5 has been reported to inhibit type I interferon production by cleaving NEMO at multiple sites, suggesting that disruption of IFN-mediated innate immunity is an important component of FIPV immune evasion [10]. Transcriptomic studies using CRFK cells and feline macrophages further indicate that FIPV infection induces distinct viral and immune-related gene-expression programs in permissive epithelial-like cells and pathogenesis-relevant macrophages [11,12,13]. These findings support the rationale for identifying molecules that can restore or amplify feline innate antiviral responses during FIPV infection.

Interferon-stimulated gene 15 (ISG15) is a ubiquitin-like modifier that plays multifaceted roles in antiviral defense, inflammatory regulation and host damage control [6]. ISG15 can be conjugated to target proteins through ISGylation, thereby regulating protein stability and function, while its free from can modulate intracellular signaling or act extracellularly as a cytokine-like molecule [11]. Structurally, ISG15 consists of two ubiquitin-like β-grasp domains, providing conserved surfaces for interactions with host and viral proteins [14,15]. Importantly, ISG15 is not simply a downstream marker of interferon activation; it can directly participate in antiviral restriction and immune regulation. For example, ISG15 has been shown to participate in MDA5-mediated antiviral signaling against coronaviruses [16,17], underscoring its role as a critical regulatory hub in antiviral immunity that integrates interferon signaling and modulates inflammatory responses [18], underscoring its potential as a therapeutic target [6,16,18,19].

Natural bioactive peptides provide a valuable reservoir for the discovery of antiviral and immunomodulatory lead compounds [20,21]. Animal-derived bioactive molecules have provided a valuable source of pharmacologically active peptides and proteins. The endogenous bioactive substances secreted by wild animals such as snakes, spiders, scorpions and centipedes are increasingly recognized as evolutionarily optimized reservoirs of bioactive peptides with therapeutic potential [20,21,22,23]. Accordingly, medicinal insects can be viewed as an underexplored branch of animal-derived peptide resources, with potential to yield structurally diverse molecules that regulate infection-associated immune and inflammatory processes [5,18]. The American cockroach, Periplaneta americana (*P. americana*), represents one such peptide-rich resource. Transcriptomic studies have identified numerous antimicrobial peptide candidates from *P. americana*, several of which exhibit potent antimicrobial activity with limited cytotoxicity [24,25]. In addition, *P. americana*-derived defensin-like peptides have shown antiviral potential in experimental models, while periplanetasin-family peptides have been reported to possess anti-inflammatory activity [26,27]. These findings suggest that *P. americana* may contain peptide molecules capable of engaging mammalian antiviral or inflammatory pathways. However, whether *P. americana*-derived peptides can target host ISG15-associated antiviral immunity and suppress FIPV infection has not been investigated.

In the present study, we identified a *P. americana*-derived peptide that inhibits FIPV infection by engaging host ISG15-associated antiviral immunity. Using molecular docking against host ISG15 as a screening strategy, we selected four candidate peptides with relatively favorable predicted binding profiles. Functional screening in Crandell Rees feline kidney (CRFK) cells identified one lead peptide with the strongest anti-FIPV activity and limited cytotoxicity. Mechanistically, the lead peptide enhanced the antiviral state of FIPV-infected CRFK cells, as evidenced by increased expression of IFNA1, IFNB, ISG15 and MX1. Furthermore, in a feline infection model, treatment with the lead peptide reduced viral burden and alleviated inflammatory injury, supporting its dual antiviral and immunomodulatory activity in vivo.

Collectively, our findings reveal a previously unrecognized antiviral function of a *P. americana*-derived peptide against FIPV and provide evidence that ISG15-linked innate immunity is amenable to pharmacological augmentation by insect-derived molecules. By expanding the functional landscape of insect peptide resources, this study provides a host-directed approach to combat FIPV infection and its associated inflammatory pathology.

## 2. Materials and Methods

### 2.1. Cells and Viruses

Crandell–Rees feline kidney (CRFK) cells were routinely maintained in our laboratory and cultured in Dulbecco’s Modified Eagle Medium (DMEM; Gibco, Waltham, MA, USA) supplemented with 10% Fetal Bovine Serum (FBS; ExCell Bio, Suzhou, China) and 100 U/mL penicillin–streptomycin. Cells were incubated at 37 °C with 5% CO_2_ humidified conditions via regular subculture.

The feline infectious peritonitis virus (FIPV) strain BJ-01 was stored in our laboratory [28]. Viral amplification was performed in CRFK cells. At 72 h post-infection, obvious cytopathic effect (CPE) was observed in the cells. Viruses were released from host cells by three cycles of freeze–thawing, followed by centrifugation at 3000× *g* for 10 min at 4 °C. The clarified supernatant was collected, aliquoted, and stored at −80 °C. To ensure stable virulence and titers, the viral stock was subjected to 3 consecutive low-passage propagations. The viral titers were determined by the TCID_50_ method.

### 2.2. Peptides Acquisition

Periplaneta americana purchased from Heyuan Meixian Breeding Base (Kunming, China) was weighed and lysed on ice in 1 mL pre-cooled lysis buffer. (0.5 M Tris–HCl, pH 8.0; 5 mM EDTA, pH 8.0; 150 mM NaCl; 10 mM MgCl_2_; 2% *v*/*v* 2-ME and a protease inhibitor cocktail). After incubation at 4 °C for 30 min with gentle rotation, the sample was centrifuged at 12,000 rpm for 15 min at 4 °C [29]. The supernatant was collected by excluding the upper lipid layer and bottom insoluble precipitate, then aliquoted and stored at −80 °C as crude protein extract.

A positive clone harboring recombinant Human GST-ISG15 plasmid (Cusabio, Wuhan, China) was cultured in ampicillin-supplemented LB medium. Protein expression was induced by 1 mM IPTG at OD_600_ and reached 0.6–0.8. Bacteria were harvested and lysed by ultrasonication, and the supernatant was purified via GST agarose affinity chromatography. The eluted protein was dialyzed at 4 °C against endotoxin-free PBS (pH 7.4) using a 10 kDa MWCO dialysis bag, with dialysate replaced three times at 1 h intervals. Protein purity and molecular weight were assessed by SDS-PAGE, and concentration was determined by a BCA assay [30]. The qualified protein was aliquoted and preserved at −80 °C.

The GST pull-down assay was performed using the GST Pull-down Kit (Biolinkedin, Shanghai, China) per the manufacturer’s instructions. Briefly, 50 μL GST-tagged magnetic agarose beads were equilibrated with pull-down buffer post-magnetic separation. Purified GST-Human ISG15 and Periplaneta americana crude protein were mixed with pretreated beads, supplemented with PMSF, and adjusted to 500 μL with pull-down buffer, with free GST as the blank control. The mixture was incubated overnight at 4 °C under gentle rotation. After repeated washing with washing buffer to eliminate non-specific binding proteins, samples were subjected to SDS-PAGE and mass spectrometry for identification of interacting proteins.

### 2.3. Peptide Screening and Synthesis

Using the keywords “Human ISG15” and “Felis catus ISG15”, the amino acid sequences of Human ISG15 (NP_005092.1) and Feline ISG15 (NP_001124315.3) were downloaded from the NCBI database: “https://www.ncbi.nlm.nih.gov/ (accessed on 12 July 2025)”. Subsequently, combined with mass spectrometry-identified data and the FIPV 3CLpro protein sequence (GenBank accession No. 2678049405), interactions were predicted using AlphaFold 3 (version 3.0.2), followed by preliminary molecular docking screening with AutoDock Vina (version 1.2.6) [31].

Homology modeling of feline ISG15 was constructed via SWISS-MODEL: “https://swissmodel.expasy.org (accessed on 10 August 2025)”, with Human ISG15 (NP_005092.1) serving as the template. The quality of the resultant structural model was validated using UCLA-DOE LAB SAVES v6.1: “https://saves.mbi.ucla.edu (accessed on 10 August 2025)”. ISG15 orthologs from *Musculus*, *Canis lupus familiaris*, *Sus scrofa*, *Ovis aries*, *Bos taurus*, and *Cavia porcellus* (accessions: NP-056598.2, XP-038522193.1, NP-001121941.2, NP-001009735.1, NP-776791.1, XP-003461574.1) were subsequently obtained. Multiple-sequence alignment was performed using ESPript3: “https://espript.ibcp.fr/ESPript/ESPript/index.php (accessed on 11 August 2025)”, whereas homology assessment and phylogenetic analysis were conducted with iTOL: “https://itol.embl.de/ (accessed on 11 August 2025)” and Chiplot heatmap tool: “https://chiplot.online/heatmap.html (accessed on 11 August 2025)”. Structural visualization was implemented using PyMOL software (version 2.5.5).

Physicochemical characteristics of target proteins were analyzed through ExPASy-ProtParam: “https://web.expasy.org/protparam/ (accessed on 12 August 2025)”, and potential toxicity of candidate peptides was predicted using ToxinPred: “https://webs.iiitd.edu.in/raghava/toxinpred/design.php (accessed on 12 August 2025)”. Four candidate peptides (PI8, PI9, PI20, and PI28) were ultimately screened for subsequent validation. All synthetic peptides were manufactured by Shanghai Jill Biochemical Co., Ltd. (Shanghai, China), with purity guaranteed above 95%, and further characterized by reversed-phase high-performance liquid chromatography (RP-HPLC) and mass spectrometry.

### 2.4. Biolayer Interferometry

The interaction between the peptide and feline ISG15 was characterized using biolayer interferometry (BLI). Raw sensorgrams were collected using Octet BLI Discovery software (Pall ForteBio, Menlo Park, CA, USA), and subsequent data analysis was carried out in Octet Analysis Studio 13.0. All measurements were conducted at a constant temperature of 25 °C in Greiner 96-well HTS black microplates with a working volume of 200 μL per well. GST biosensors were pre-wetted in phosphate-buffered saline (PBS, pH 7.4) for a minimum of 10 min prior to the assay. PBST (PBS, pH 7.4) supplemented with 0.1% bovine serum albumin (BSA) and 0.1% Tween-20 was used as the assay buffer to reduce non-specific binding. Serial concentrations of the peptide were prepared to evaluate its binding to feline ISG15.

### 2.5. Cell Viability Assay

Cell viability was assessed via Super Kine^tm^ High-Sensitivity Cell Counting Kit-8 (CCK-8, Abbkine, Wuhan, China) following the manufacturer’s instructions. Briefly, CRFK cells were seeded into 96-well plates with 1.0 × 10^4^ cells per well for 12 h, then Cells were exposed to gradient concentrations of peptides for 24 h. CCK-8 reagent was added at a 1:10 ratio, followed by incubation at 37 °C for 0.5–4 h. The absorbance at 450 nm was determined using a SpectraMax M2e multi-mode microplate reader (Molecular Devices, San Jose, CA, USA) with SoftMax Pro 7.0 software.

### 2.6. Antiviral Activity of Peptides In Vitro

CRFK cells were seeded in 12-well plates at a density of 2.5 × 10^5^ cells per well. At 80–90% confluence, cells were inoculated with FIPV (MOI = 0.05) and incubated at 37 °C for 1 h for viral adsorption. After removing the viral inoculum, cells were cultured for another 24 h in maintenance medium containing gradient concentrations of peptides. Total RNA was extracted from harvested cells, and FIPV *N* gene transcription level was detected by RT- qPCR to evaluate peptide antiviral efficacy. Primer sequences are listed in Table S5.

### 2.7. Impact of PI-8 on FIPV Direct Inactivation, Adsorption, and Replication

Direct Inactivation Assay: FIPV (MOI = 0.05) was incubated with various concentrations of PI-8 at 37 °C for 1–4 h. The mixture was diluted 200-fold to quench peptide activity and inoculated into CRFK cells. Intracellular FIPV *N* gene expression was detected to evaluate PI-8’s direct inactivation effect on viral particles.

Adsorption Inhibition Assay: FIPV (MOI = 0.05) mixed with various concentrations of PI-8 was added to CRFK cells and incubated at 4 °C for 1 h (viral adsorption only, no entry). Unbound virus and peptides were removed by cold PBS washing, and cells were harvested for RT-qPCR to evaluate adsorption inhibition.

Replication Inhibition Assay: CRFK cells were first infected with FIPV (MOI = 0.05) at 37 °C for 1 h to complete viral adsorption and entry. After washing, cells were then treated with different concentrations of PI-8 for 24 h, and viral genome replication was detected.

### 2.8. Plaque Assay

CRFK cells were seeded in 12-well plates and placed in a 37 °C under 5% CO_2_. Cells were washed with PBS and inoculated with virus (1 × 10^2^ PFU/0.1 mL per well), followed by 1 h incubation at 37 °C for viral adsorption. After PBS washing to remove unbound virus, cells were cultured with maintenance medium containing diluted PI-8 for 48 h. Cell culture supernatants were harvested to quantify viral progeny titers via plaque assay, expressed as log_10_ PFU/mL.

### 2.9. Real-Time Fluorescent Quantitative PCR Assay

Total RNA was extracted via the Trizol reagent (Thermo Fisher Scientific, Cambridge, MA, USA), RNA purity and concentration were determined using a NanoDrop One UV–Vis Spectrophotometer (Thermo Fisher Scientific, Waltham, MA, USA), reverse-transcribed into cDNA following the HiScript II RT SuperMix for qPCR (+gDNA wiper) kit (Vazyme Biotech Co., Ltd., Nanjing, China) and qPCR was performed using 2 × Universal SYBR qPCR Master Mix (Youngen Biotechnology, Beijing, China) on a real-time PCR system. The qPCR program was as follows: pre-denaturation at 95 °C for 3 min; 40 cycles of 95 °C for 15 s and 58 °C for 30 s. All Primers were synthesized by Sangon Biotech (Shanghai, China), GAPDH expression served as the reference gene for all reactions. Primer sequences are listed in Table S5.

### 2.10. Animal Experiments

A controlled experimental FIPV infection model was used to standardize viral strain, inoculation dose, infection timing, treatment initiation, and sampling schedule, thereby reducing the clinical heterogeneity associated with naturally occurring FIP cases [32]. Experimental cats were housed in a constant-temperature (24 °C) environment with sufficient food and water. To ensure experimental rigor, cats were individually caged in three fully isolated rooms and acclimatized for two weeks before the formal experiment. After acclimatization, all cats were randomly divided into three groups (*n* = 5 per group): non-infected control group, an FIPV-infected model group, or an FIPV-infected PI-8-treated group. Except for the controls, cats in the other two groups were intraperitoneally (IP) injected with 1 mL of FIPV at 10^5^·^25^ TCID_50_. After infection, blood was collected from the forearm vein every two days to dynamically monitor viral load, and saliva and anal swab samples were collected daily. Intervention was initiated immediately on day 3 post-infection when a stable increase in serum viral load was confirmed. Cats in the treatment group received daily subcutaneous (SC) injections of PI-8 (2 mg/kg), while the FIPV-infected group received an equal volume of sterile PBS. The intervention period was two weeks [32,33].

During the experiment, clinical symptoms were observed and scored every 24 h, including body weight, body temperature in the inner thigh skin, appetite, mental state, ocular and nasal discharge, fecal excretion, and neurological signs [34]. To minimize suffering, cats were euthanized at the end of the experiment in accordance with animal welfare and ethical requirements. Tissue samples (heart, liver, spleen, lung, kidney, and intestinal segments) were collected for subsequent detection and histopathological analysis.

### 2.11. Detection of Viral Load and Inflammatory Factors in Tissues

To accurately quantify the viral copy number in tissues, the standard plasmid stored in the laboratory was serially diluted 10-fold (concentration range: (10^1^–10^9^ copies/μL) and used as the template for qPCR amplification. A standard curve was established based on the Ct values and the logarithm of the template concentrations.

Tissues were homogenized using a tissue grinder (Shanghai Jingxin Industrial Development Co., Ltd., Shanghai, China), and total RNA was extracted using the Trizol method and reverse-transcribed into cDNA. The copy number of the FIPV-N gene in each tissue was quantified by qPCR. Meanwhile, the transcription levels of key inflammatory factors (TNF-α, IL-1β, IL-6, IL-1A) were detected using corresponding primers (Table S5).

### 2.12. Histopathological Examination (H&E Staining)

Tissues were fixed in 4% paraformaldehyde for 72 h, followed by paraffin embedding, pathological sectioning, and hematoxylin-eosin (H&E) staining performed by Servicebio Biotechnology Co., Ltd., (Wuhan, China).

### 2.13. Ethical Statement

A standardized experimental FIPV infection cat model was established for the initial controlled proof-of-concept in vivo test of PI-8. Naturally occurring FIP cases exhibit substantial heterogeneity in disease stage, viral burden, prior treatment, co-infection status, supportive care, and timing of treatment initiation, which would make it difficult to determine whether the observed antiviral and pathological effects were attributable to PI-8 itself. This experimental infection model enables standardization of viral strain, inoculation dose, infection route, infection timing, treatment initiation and sampling schedule to eliminate confounding clinical variables.

All animal operations were reviewed and approved by the Experimental Animal Ethics Committee of Shanxi Agricultural University (Approval No. SXAU-EAW-2025C. JR.012009473, approved on 10 December 2025). Animal housing, viral inoculation and sample collection strictly followed the international 3R principles (Replacement, Reduction, Refinement) and the ARRIVE guidelines. We reasonably minimized the number of experimental animals, optimized all operative procedures, and defined explicit humane endpoints to minimize animal pain and distress throughout the trial. Protocols for animal housing and daily care fully complied with the requirements specified in Regulations for the Administration of Affairs Concerning Experimental Animals issued by the Ministry of Science and Technology of China. Meanwhile, future studies in naturally infected client-owned cats will be necessary for clinical validation of PI-8.

### 2.14. Statistical Analysis

All statistical analyses were performed using GraphPad Prism 11.0 software. Data are presented as mean ± SD (*n* ≥ 3). Multiple-group comparisons were analyzed by one-way or two-way analysis of variance (ANOVA). Statistical significance was defined as * *p* < 0.05, ** *p* < 0.01, *** *p* < 0.001, and ns, not significant.

## 3. Results

### 3.1. ISG15-Targeted Screening Yields Four P. americana Peptides

**Figure 1:**
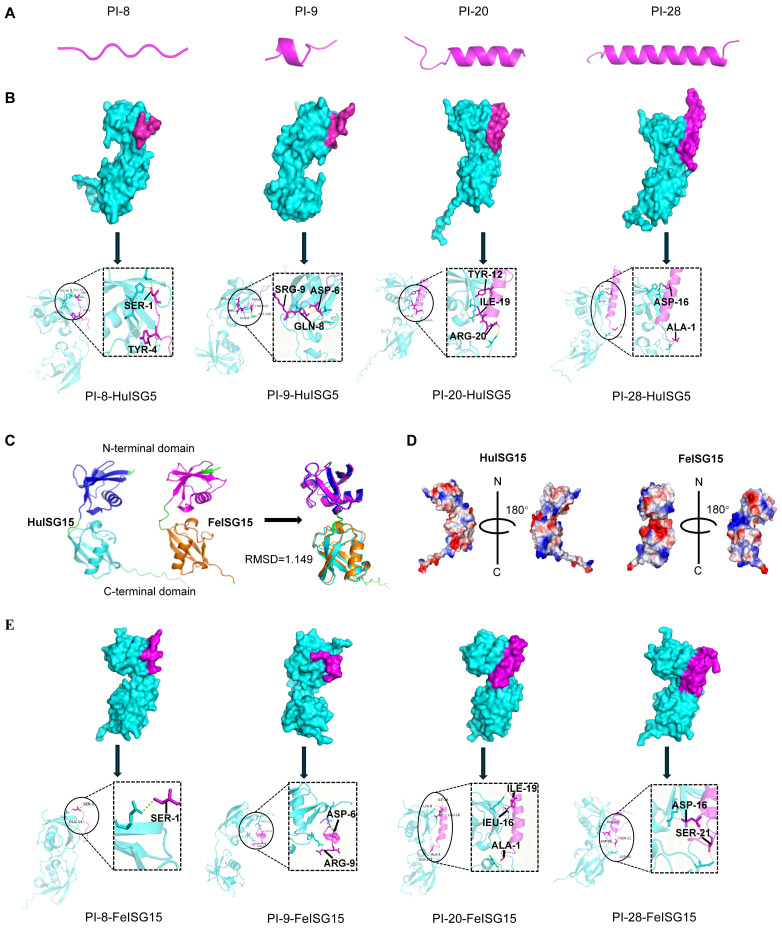
Structural modeling identifies four *P. americana* peptides with predicted ISG15-binding potential. (**A**) Predicted secondary structures of the four candidate peptides. (**B**) Predicted docking models of PI-8, PI-9, PI-20 and PI-28 with human ISG15 (bule). Enlarged views indicate key amino acid residues at the predicted peptide–ISG15 interfaces. (**C**) Structural superposition of human ISG15 and modeled feline ISG15. The calculated RMSD was 1.149 Å. (**D**) Molecular surface electrostatic potential maps of human and feline ISG15 (bule). (**E**) Predicted docking models of PI-8, PI-9, PI-20 and PI-28 with feline ISG15. Enlarged views indicate key amino acid residues at the predicted peptide–ISG15 interfaces.

To identify *P. americana*-derived peptides with potential activity toward the host ISG15-associated antiviral axis, we selected human ISG15 as the initial screening target because its experimentally resolved structure is well characterized and provides a representative mammalian ISG15 model. Recombinant human ISG15 was used as bait in pull-down assay with *P. americana* extract leading to the identification of 203 ISG15-associated peptide fragments (Supplementary Material Table S1). These peptides were then subjected to AlphaFold3-based interaction prediction and AutoDock Vina docking against human ISG15. Among the recovered peptide fragments, four candidates, designated PI-8, PI-9, PI-20 and PI-28, showed favorable predicted interaction profiles with human ISG15 and were selected for further analysis (Figure 1A,B). Docking models showed that these peptides occupied surface-exposed regions of human ISG15 and formed local interaction networks involving hydrogen bonds and electrostatic contacts (Figure 1A,B). Their sequences, molecular masses, source protein accessions, charge states, docking scores, binding energies, instability indices and predicted toxicity are summarized in Table S2.

Because FIPV validation was performed in feline cells and cats, we next assessed the conservation between human and feline ISG15. Pairwise sequence alignment showed that human and feline ISG15 shared substantial sequence similarity (Figure S1A), and a multi-species ISG15 homology heatmap further showed that the identity between human ISG15 and feline, with 70.06%, was higher than that between human ISG15 and most other species examined (Figure S1B). Structural superposition further showed that feline ISG15 preserved the two-domain ubiquitin-like fold of human ISG15, with a low RMSD of 1.149 Å between the modeled structures (Figure 1C). Consistently, human ISG15 and feline ISG15 displayed similar molecular surface electrostatic distributions (Figure 1D). These results strongly support the existence of relatively close conservation between the human and feline orthologs (Figure S1B). To further examine whether the selected peptides could also engage feline ISG15, PI-8, PI-9, PI-20, and PI-28 were re-docked against the modeled feline ISG15 structure. The predicted docking profiles are summarized in Table S3. All four candidates retained predicted binding to feline ISG15, and the interation sites were located on accessible surface regions comparable to those observed in the human ISG15 docking models (Figure 1E). These results supported the potential relevance of the four candidates in feline ISG15-associated antiviral studies. Both human ISG15 and feline ISG15 formed predicted contacts with FIPV protease, suggesting that the two ISG15 orthologs may share similar interaction tendencies toward this viral protein (Figure S2). Collectively, these findings indicate that the sequence and structural conservation between human and feline ISG15 supports the use of human ISG15-based screening results to further test selected peptides in the feline FIPV model.

### 3.2. PI-8 Is Identified as the Lead ISG15-Binding Anti-FIPV Peptide

**Figure 2:**
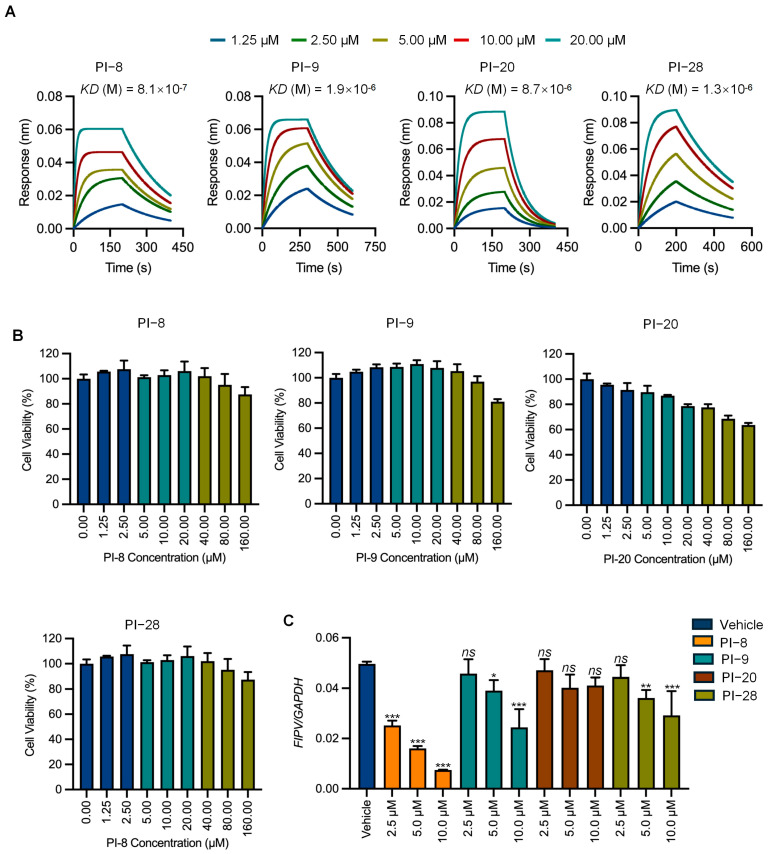
BLI validation and anti-FIPV screening identify PI-8 as the lead peptide from four *P. americana* peptides. (**A**) Biolayer interferometry analysis of the binding between recombinant feline ISG15 and the indicated candidate peptides. (**B**) Cell viability of CRFK cells treated with increasing concentrations of the indicated candidate peptides, as determined by CCK-8 assay. (**C**) qPCR analysis of FIPV RNA expression levels in infected CRFK cells treated with the indicated peptides. PI-8 displayed the strongest suppression of FIPV RNA accumulation and was selected for subsequent functional and mechanistic studies. The data represent three independent experiments and are presented as the mean ± SD. Statistical significance was determined by one-way ANOVA with multiple-comparison test. * *p* < 0.05, ** *p* < 0.01, *** *p* < 0.001; ns, not significant (**C**).

To experimentally validate the predicted interaction between the candidate peptides and feline ISG15, we used recombinant feline ISG15. All four peptides bound feline ISG15 in a concentration-dependent manner, with PI-8 showing the strongest binding among the candidates (*KD*(M) = 8.1 × 10^−7^; Figure 2A). To prioritize candidate peptides for the functional validation, we first assessed the cytotoxicity of the four peptides in CRFK cells using a CCK-8 assay. With the exception of PI-20, treatment with the candidate peptides within the concentration range of 0–40 μM did not significantly affect cell ability, indicating that this concentration range is suitable for antiviral screening (Figure 2B). We then infected CRFK cells with FIPV and treated with candidate peptides (0–10 μM). Viral replication was quantified by qPCR analysis of the FIPV gene at 24 h post-infection. Among these four peptides, PI-8 induced the most significant reduction in FIPV RNA levels in a concentration-dependent manner (Figure 2C). Together, the BLI and cell-based screening results identified PI-8 as the lead candidate, combining the strongest feline ISG15-binding response with the most potent anti-FIPV activity and limited cytotoxicity in CRFK cells. PI-8 was therefore selected for subsequent functional and mechanistic analyses.

### 3.3. PI-8 Inhibits Post-Entry FIPV Replication and Induces IFN-ISG15 Responses

**Figure 3:**
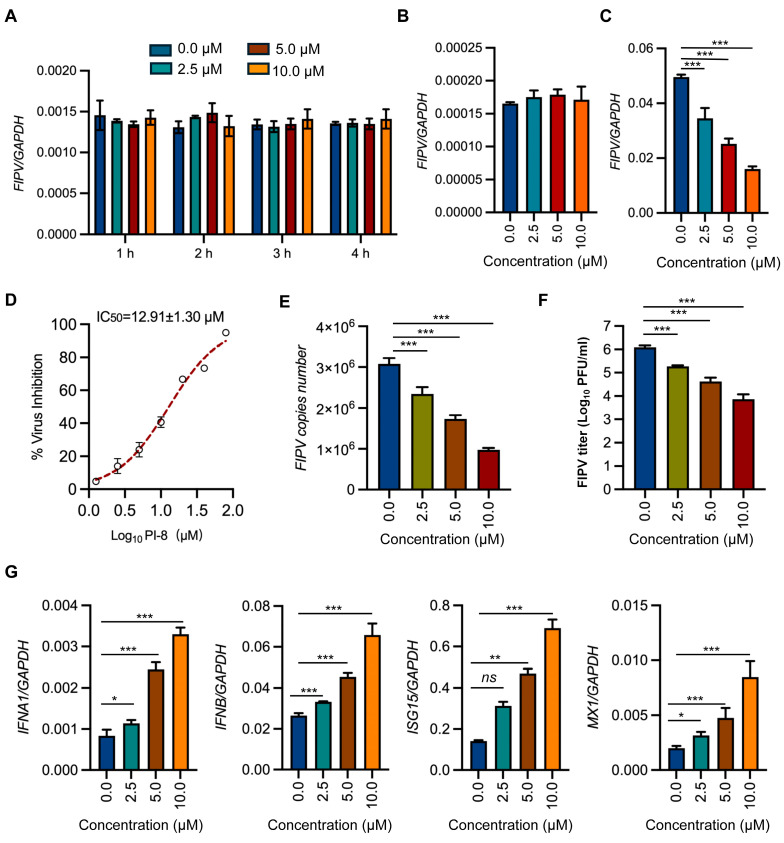
PI-8 inhibits FIPV replication and induces IFN-ISG15 responses in CRFK cells. (**A**–**C**) The impact of PI-8 on the direct inactivation, adsorption, and replication of stages of FIPV. For the assessment of direct inactivation, FIPV (MOI = 0.05) was pre-incubated with PI-8 at 37 °C for 1, 2, 3, or 4 h before infection of CRFK cells, after 24 h incubation at 37 °C, RNA was extracted for RT-qPCR detection of viral RNA. For adsorption inhibition, FIPV (MOI = 0.05) and PI-8 were added simultaneously to cells and incubated at 4 °C for 1 h, followed by RT-qPCR analysis. To evaluate replication inhibition, CRFK cells were infected with FIPV (MOI = 0.05) for 1 h, and subsequently treated with increasing concentrations of PI-8 for 24 h, after which viral RNA was quantified by RT-qPCR. (**D**) Dose–response curve of PI-8-mediated inhibition of FIPV replication. The IC_50_ value was calculated as 12.91 ± 1.30 μM. (**E**) Absolute quantification of FIPV-*N* gene copy numbers in FIPV-infected CRFK cells treated with the indicated concentrations of PI-8. (**F**) Viral titers meaured by plaque assay following treatment with the indicated concentrations of PI-8. (**G**) CRFK cells were infected with FIPV and then treated with various concentrations of PI-8 for 24 h. The expression levels of IFNA1, IFNB, ISG15, and MX1 were determined by qPCR. The data represent three independent experiments and are presented as the mean ± SD. Statistical significance was determined by one-way ANOVA with multiple-comparison test. * *p* < 0.05, ** *p* < 0.01, *** *p* < 0.001; ns, not significant (**C**,**E**–**G**).

To define the step of the FIPV life cycle affected by PI-8, we assessed its activity during viral direct inactivation, adsorption and replication. PI-8 had little effect on FIPV RNA levels; viral particles were pre-incubated with PI-8 and then added to cells, indicating that it did not directly inactivate extracellular virions (Figure 3A). Likewise, treatment with PI-8 during the adsorption period had no significant effect on FIPV RNA abundance (Figure 3B). In contrast, addition of PI-8 after viral adsorption markedly reduced intracellular FIPV RNA levels in a concentration-dependent manner (Figure 3C). These results indicate that PI-8 acts mainly at a post-entry stage of FIPV infection, consistent with an inhibitory effect on viral replication rather than direct virion inactivation or blockade of viral adsorption. The antiviral potency of PI-8 was then quantified by dose–response analysis. PI-8 suppressed FIPV replication in CRFK cells with an IC_50_ value of 12.91 ± 1.30 μM (Figure 3D). Absolute quantification of the FIPV-N gene further confirmed a concentration-dependent decrease in viral RNA copies number following PI-8 treatment, and PI-8 had more than 50% inhibition of FIPV infection at 10 μM (Figure 3E). Consistently, plaque assay analysis showed that PI-8 significantly decreased infectious FIPV titers in a dose-dependent manner, demonstrating that PI-8 reduced not only viral RNA accumulation but also the production of infectious progeny virus (Figure 3F).

Because PI-8 was identified from an ISG15-directed docking screen, we next examined whether its antiviral activity was accompanied by changes in the IFN–ISG15 transcriptional response. PI-8 treatment significantly regulated the expression of IFNA, IFNB, and ISG15 in a dose-dependent manner (Figure 3G), while MX1 was also upregulated as a canonical antiviral ISG readout (Figure 3G). Taken together, these results show that PI-8 predominantly inhibits the post-entry replication phase of FIPV infection and promotes an IFN-ISG15-associated antiviral transcriptional response in feline cells.

### 3.4. PI-8 Reduces Systemic and Tissue Viral Loads in FIPV-Infected Cats Without Affecting Their Body Weight and Temperature

**Figure 4:**
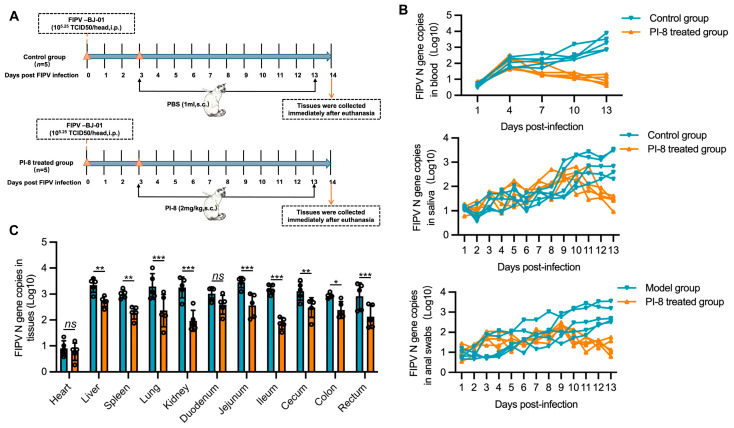
PI-8 reduces viral burden in FIPV-infected cats. (**A**) The experimental schedule of PI-8 treatment and FIPV inoculation for cats. (**B**) Viral loads in blood, saliva and anal swabs from control and PI-8-treated cats were monitored over time by qPCR. (**C**) Endpoint viral burdens in major organs and intestinal tissues collected were determined by qPCR (*n* = 5), Circles represent individual cats. Data are shown as mean ± SD. Statistical significance was determined by two-way Anova, * *p* < 0.05, ** *p* < 0.01, *** *p* < 0.001, ns, not significant (**C**).

To assess the antiviral efficacy of PI-8 in vivo, fifteen female cats were enrolled and assigned to a non-infected control group, an FIPV-infected model group, or an FIPV-infected PI-8-treated group, with five cats in each group; detailed information for each cat is provided in Table S4. Cats challenged intraperitoneally with FIPV and then treated with PI-8 at 2 mg/kg or PBS according to the experimental schedule are shown in Figure 4A. Viral loads were in blood, saliva and anal swabs during infection, and tissues were collected for FIPV copies number quantification at the end of the experiment. In the infected control group, viral loads in blood increased progressively over time. PI-8 treatment markedly reduced blood viral RNA levels during the middle and late stages of infection (Figure 4B). A similar trend was observed in saliva and anal swab samples, with PI-8-treated cats exhibiting lower viral shedding than controls, particularly at later time points (Figure 4B). In agreement with the viral load kinetics observed in peripheral samples, analysis of tissues collected showed that PI-8 treatment reduced FIPV RNA accumulation in multiple organs, including the liver, spleen, lung, kidney, jejunum, ileum, cecum, colon and rectum, whereas viral loads in the heart and duodenum were not significantly altered (Figure 4C). Thus, PI-8 reduced both systemic viral burden and tissue viral accumulation in FIPV-infected cats.

Body weight and body temperature were monitored daily to evaluate the general condition of cats. Body weight remained largely stable in the control, FIPV-infected and PI-8-treated groups, with no sustained weight loss observed during the 14-day observation period (Figure S3A). Body temperature also remained within a narrow range in all groups, and neither FIPV infection nor PI-8 treatment caused persistent fever or hypothermia (Figure S3B). These data indicate that, under the present experimental conditions, FIPV infection and PI-8 administration did not produce overt changes in body weight or body temperature.

### 3.5. PI-8 Alleviates FIPV-Induced Pathology and Inflammation in Cats

**Figure 5:**
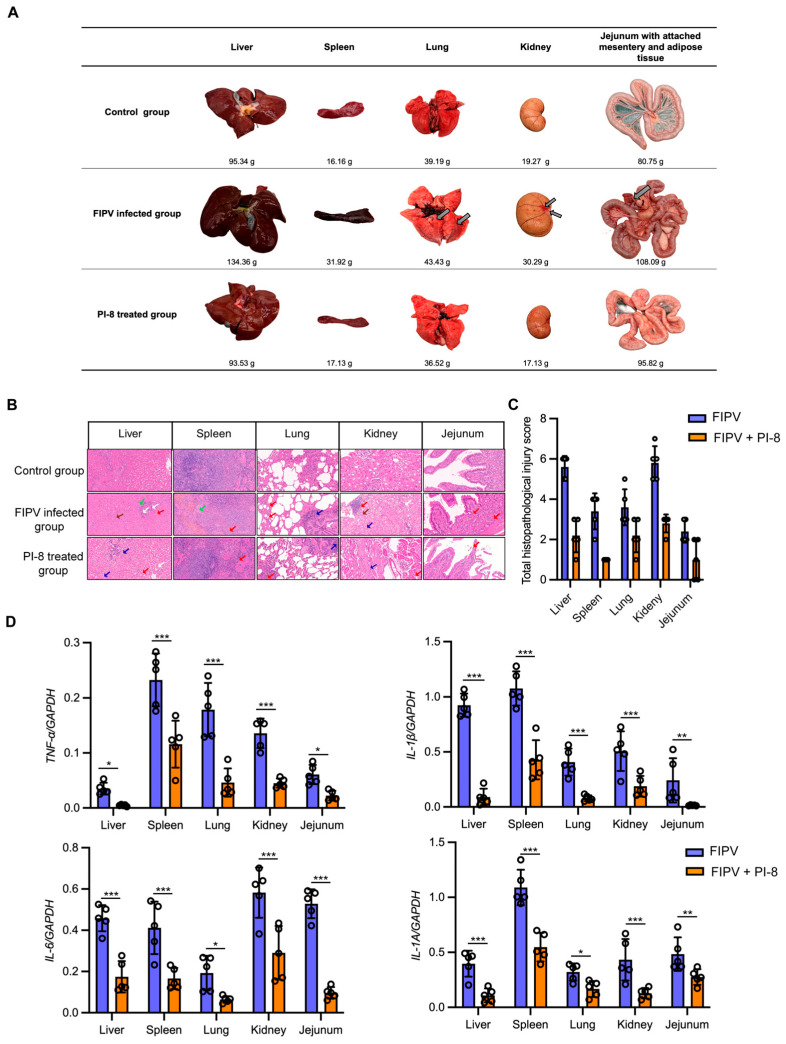
PI-8 alleviates pathology and inflammation in FIPV-infected cats. (**A**) Representative images of the liver, spleen, lung, kidney, and jejunum tissues from non-infected control, FIPV-infected model, PI-8-treated cats. (**B**) Representative H&E-stained sections of the indicated tissues showing histopathological changes after FIPV infection and PI-8 treatment, Different colored arrows indicate distinct histopathological alterations. Scale bars, 50 μm. (**C**) Quantification of total histopathological injury scores in the liver, spleen, lung, kidney, and jejunum from FIPV-infected and PI-8-treated cats (*n* = 5), Circles represent individual cats. (**D**) qPCR analysis of inflammatory cytokine expression in tissues from FIPV-infected and PI-8-treated cats. mRNA levels of TNF-α, IL-1β, IL-6, and IL-1A were normalized to GAPDH (*n* = 5), Circles represent individual cats. Data are shown as mean ± SD. Statistical significance was determined by two-way Anova. * *p* < 0.05, ** *p* < 0.01, *** *p* < 0.001 (**C**,**D**).

To further evaluate whether the reduction in viral burden was accompanied by attenuation of tissue injury, major organs and intestinal tissues were examined. Compared with the non-infected control group, FIPV-infected cats showed prominent gross lesions, including hepatic and splenic enlargement with congestion, pulmonary and renal congestion or edema, and intestinal swelling with mesenteric involvement (Figure 5A). These pathological changes were visibly attenuated in PI-8-treated cats (Figure 5A). Histopathological analysis further confirmed the protective effect of PI-8. HE staining revealed marked tissue lesions in FIPV-infected cats, including inflammatory cell infiltration, congestion, edema, and disruption of normal tissue architecture in the liver, spleen, lung, kidney, and jejunum (Figure 5B). In contrast, PI-8-treated cats displayed milder histological alterations in these tissues. Consistently, quantitative histopathological scoring showed that PI-8 significantly reduced the total injury scores in all examined tissues compared with the FIPV-infected group (Figure 5C). qPCR analysis further showed that PI-8 treatment reduced the expression of inflammatory cytokines in FIPV-infected tissues. Compared with the FIPV group, PI-8 significantly decreased TNF-α, IL-1β, IL-6, and IL-1A expression in the liver, spleen, lung, kidney, and jejunum, although the magnitude of reduction varied among tissues (Figure 5D). These results indicate that PI-8 attenuates FIPV-associated pathological injury and inflammatory responses across multiple organs.

## 4. Discussion

Feline infectious peritonitis remains a challenging coronavirus-associated disease because its progression is driven by both viral replication and dysregulated host immunity. Direct-acting antivirals, including the nucleoside analog GS-441524 and the protease inhibitor GC376, have demonstrated that FIPV is pharmacologically tractable and that suppression of viral replication can markedly improve disease outcome [32,35,36]. However, FIP is also characterized by systemic dissemination, monocyte/macrophage-associated infection, vasculitis, serositis and pyogranulomatous inflammation [35,37,38]. These features indicate that molecules with both antiviral and immune-modulatory properties may provide additional value. In this study, we identified PI-8, a *P. americana*-derived peptide, as a candidate molecule that suppresses FIPV replication and modulates infection-associated immune responses.

PI-8 was initially identified through ISG15-associated peptide enrichment and computational prioritization. ISG15 is an interferon-inducible ubiquitin-like protein involved in antiviral defense, ISGylation, inflammatory regulation and host damage control [11,39,40]. Human ISG15 was used for the first enrichment step because its structure is well characterized. The subsequent sequence and structural comparison between human and feline ISG15 supported further evaluation of the selected peptides in feline systems. Although the BLI assays confirmed direct binding between PI-8 and recombinant feline ISG15, these data do not establish that is required for the antiviral activity of PI-8. Further studies using ISG15 loss-of-function approaches, together with residue mutational analysis, will be needed to define the functional and molecular relationship between PI-8 and ISG15. The predicted binding poses of PI-8 with human and feline ISG15 showed differences in the potential contribution of key peptide residues, including S1 and Y4. Further residue-level mutagenesis may help define the peptide–ISG15 interface and guide future optimization of PI-8 for improved antiviral efficacy, stability, and in vivo delivery.

Among the candidate peptides, PI-8 showed the strongest anti-FIPV activity in CRFK cells with limited cytotoxicity. Time-of-addition experiments indicated that PI-8 did not mainly inactivate extracellular virions or block viral adsorption, but reduced viral RNA accumulation after entry. This activity profile differs from compounds with direct virucidal or entry-blocking effects and suggests that PI-8 acts during the intracellular phase of FIPV infection. Previous studies have shown that natural compounds may inhibit FIPV at different stages of the viral life cycle, including attachment, penetration, post-entry replication and direct virion inactivation [28,41]. PI-8 therefore adds to the range of natural-product-derived molecules with potential anti-FIPV activity.

A key finding of this study is that PI-8 treatment was associated with increased expression of IFNA1, IFNB, ISG15 and MX1 in FIPV-infected CRFK cells. This observation links the ISG15-associated selection of PI-8 with an antiviral transcriptional response in feline cells. Coronaviruses, including FIPV, can antagonize type I interferon induction and downstream antiviral signaling; FIPV Nsp5 has been reported to suppress type I interferon production by cleaving NEMO [42]. Thus, PI-8 may help reinforce an IFN–ISG15-related antiviral state during FIPV infection. However, whether ISG15 functions as a direct mediator of PI-8 activity remains to be determined.

The in vivo data further support the antiviral and immune-regulatory activity of PI-8. PI-8 reduced FIPV RNA levels in blood, saliva, anal swabs and multiple tissues, indicating reduced systemic viral burden and viral shedding. In parallel, PI-8 alleviated gross pathological lesions, reduced histopathological injury scores and decreased the expression of TNF-α, IL-1β, IL-6 and IL-1A in affected tissues. These findings suggest that PI-8 not only limits viral RNA accumulation but also attenuates the inflammatory tissue environment associated with FIPV infection. Because excessive inflammatory responses are central to FIP-associated injury [35,38,42], this dual antiviral and immune-modulatory profile may be biologically relevant. Although naturally infected client-owned cats will be important for future clinical validation, the experimental model used here enabled a controlled preliminary evaluation of PI-8 during acute FIPV infection.

The activity of PI-8 may reflect broader properties of animal-derived functional peptides. Bioactive peptides can interact with host proteins, modulate signaling thresholds and reshape innate immune responses. *P. americana* is an underexplored source of such molecules. Transcriptomic studies have identified multiple antimicrobial peptide candidates from *P. americana*, and previous reports have described antiviral or anti-inflammatory activities of *P. americana*-derived defensin-like and periplanetasin-family peptides [43,44,45]. The present study extends these observations by showing that a *P. americana*-derived peptide can inhibit FIPV infection in feline cells and cats while also modulating antiviral and inflammatory responses.

Several limitations remain. First, although BLI assays confirmed direct binding between PI-8 and recombinant feline ISG15, the functional requirement of ISG15 for PI-8-mediated antiviral activity remains to be established. Future studies using ISG15 knockdown, overexpression, or rescue approaches, combined with residue-level mutational analysis, will be needed to clarify the functional dependence and molecular interface between PI-8 and ISG15. Second, although CRFK cells are useful for FIPV propagation and antiviral screening, they do not fully reproduce macrophage-centered FIP pathogenesis. Future work should test PI-8 in feline macrophages, monocyte-derived macrophages or primary immune cells. Third, the in vivo findings should be interpreted as preliminary evidence of acute-phase anti-FIPV activity. The use of five cats per group was determined under animal ethical considerations and the principle of minimizing animal use while allowing assessment of treatment-associated effects. In addition, the 14-day monitoring schedule was adopted based on the established FIPV infection model described in [32], which focuses on acute-phase infection outcomes. Therefore, larger animal cohorts, longer observation periods, and additional dosing regimens will be required to further evaluate the long-term therapeutic potential of PI-8. Fourth, pharmacokinetics, tissue distribution, serum stability, immunogenicity, optimal dosing and long-term safety require further evaluation. Despite its anti-FIPV activity in vitro and in vivo, PI-8 remains at an early stage of peptide-based therapeutic development. Further studies are needed to evaluate its serum stability, pharmacokinetic profile, biodistribution, delivery route, and dosing regimen before its translational potential can be fully assessed [46,47,48,49]. It will also be important to determine whether PI-8 has additive or complementary effects with established antivirals such as GS-441524 or protease inhibitors.

In summary, PI-8 is a *P. americana*-derived peptide with dual anti-FIPV and immune-regulatory activity. It suppresses post-entry viral replication, enhances an IFN–ISG15-associated antiviral response, reduces systemic and tissue viral loads, and lessens inflammatory injury in infected cats. Given the preliminary nature of the animal study, further validation in larger and longer-term in vivo studies is warranted before its therapeutic potential can be fully defined. This study highlights the potential of insect-derived functional peptides as dual-acting antiviral and immunomodulatory resources for FIP and other veterinary coronavirus diseases, while further pharmacological optimization will be required for peptide-based therapeutic translation.

## 5. Conclusions

Feline infectious peritonitis virus (FIPV) infection causes severe tissue damage driven by both viral replication and aberrant inflammation. While direct-acting antivirals suppress replication, dual-functional agents are urgently needed to mitigate inflammatory pathology. Here, we identified PI-8, an ISG15-binding bioactive peptide derived from Periplaneta americana. In vitro, PI-8 exhibited negligible cytotoxicity and potently inhibited FIPV replication at the post-entry stage, while concurrently upregulating type I interferons and downstream ISGs (ISG15 and MX1) to counteract FIPV-mediated IFN antagonism. In vivo, PI-8 significantly reduced viral loads in physiological specimens and visceral organs, alleviated histopathological lesions, and suppressed proinflammatory cytokine overexpression in FIPV-infected cats. Despite these promising results, the precise binding kinetics between PI-8 and feline ISG15, alongside the peptide’s pharmacokinetic profiles, safety margins, and synergistic efficacy with established drugs like GS-441524, warrant further biochemical validation. Collectively, our findings delineate PI-8 as a promising host-directed therapeutic candidate for FIPV and related veterinary coronaviral diseases.

## Data Availability

Data will be made available on request.

## Supplementary Materials

The following supporting information can be downloaded at: https://www.mdpi.com/article/10.3390/biology15151324/s1, Figure S1: Sequence and structural comparison of ISG15 orthologs; Figure S2: Predicted docking of human and feline ISG15 with FIPV protease. Predicted docking models of human and feline ISG15 with FIPV protease, with key interface residues highlighted; Figure S3: Body weight and body temperature of cats remain stable during FIPV infection and PI-8 treatment; Table S1: ISG15 pull-down identified peptides from *P. americana* extract; Table S2: Four *P. americana* peptides information and docking results with human ISG15; Table S3: Four *P. americana* peptides information and docking results with Feline ISG15; Table S4: Information of cats; Table S5: The sequences of primers for RT-qPCR.
