# Supplementary material for: A Natural Peptide from the American Cockroach Suppresses Feline Infectious Peritonitis Virus
Source: Biology (Basel). 2026 Aug 6;15(15):1324. doi: 10.3390/biology15151324 (PMC13464918; doi:10.3390/biology15151324)
Supplement: Supplementary file 1 [file biology-15-01324-s001.zip › Supplementary Materials.pdf]

Article

A natural peptide from the American cockroach suppresses feline infectious peritonitis virus

Tengyu Zhu †, Jinfeng Hou †, Tong Jing †, Yibo Wu, Rui Meng, Ruobing Liu, Kun Yuan, Sunchengai Cao, Xinyan Yang, Jiayi Wang, Ziyin Wang, Zhengyan Zhu, Yaogui Sun * and Lin Jin *

Shanxi Key Laboratory for Modernization of TCVM, College of Veterinary Medicine, Shanxi Agricultural University, Taiyuan 030031, China; zhutengyu@sxau.edu.cn (T.Z.); 2024308950@stu.sxau.edu.cn (J.H.); 20222017319@stu.sxau.edu.cn (T.J.); 2024204300@stu.sxau.edu.cn (Y.W.); 2024309190@stu.sxau.edu.cn (R.M.); 2024204040@stu.sxau.edu.cn (R.L.); 20233841@stu.sxau.edu.cn (K.Y.); 20232017024@stu.sxau.edu.cn (S.C.); 20232017004@stu.sxau.edu.cn (X.Y.); 20241312032@stu.sxau.edu.cn (J.W.); 20252018411@stu.sxau.edu.cn (Z.W.); 20252018518@stu.sxau.edu.cn (Z.Z.)

* Correspondence: sunyaogui@sxau.edu.cn (Y.S.); jinlin2021@sxau.edu.cn (L.J.)

† These authors contributed equally to this work.

Supplementary material

Figure S1. Sequence and structural comparison of ISG15 orthologs.

Figure S2. Predicted docking of human and feline ISG15 with FIPV protease.

Figure S3. Body weight and body temperature of cats remain stable during FIPV infection and PI-8 treatment.

Table S1. ISG15 pull-down identified peptides from *P. americana* extract.

Table S2. Four P. americana peptides information and docking results with human ISG15.

Table S3. Four P. americana peptides information and docking results with Feline ISG15.

Table S4. Information of cats

Table S5. The sequences of primers for RT-qPCR

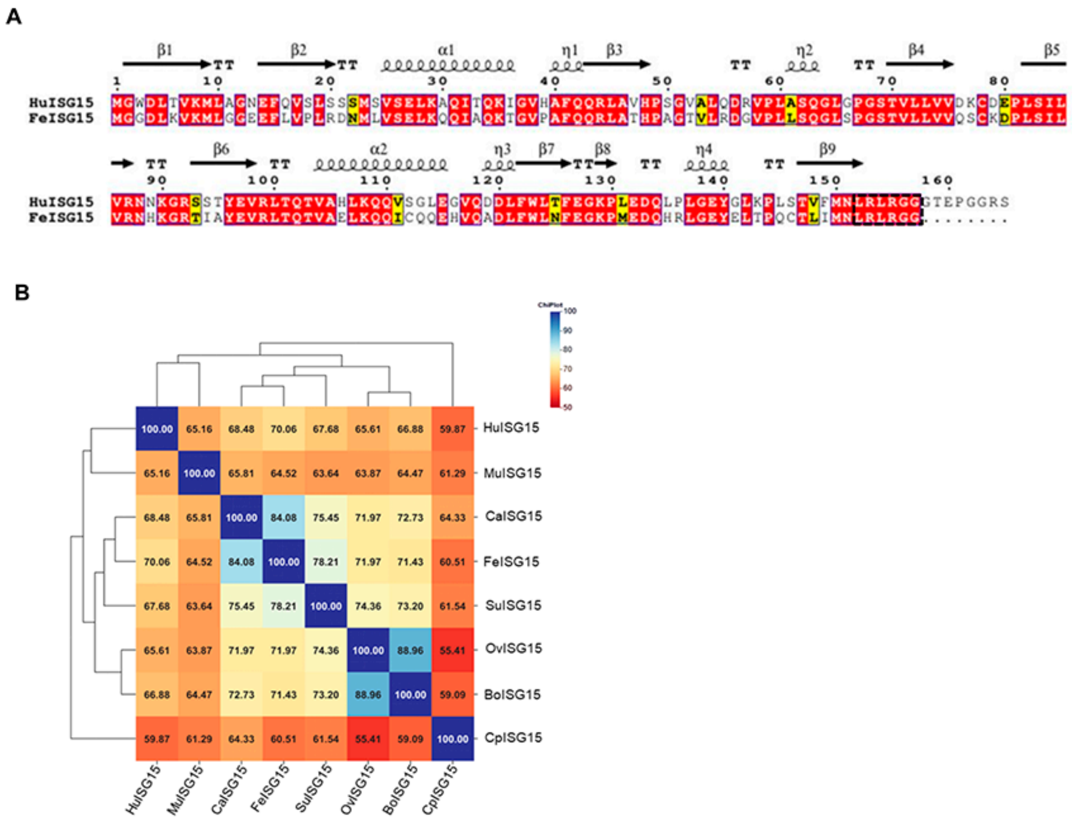

Figure S1. Sequence and structural comparison of ISG15 orthologs. **(A)** Pairwise sequence alignment of human and feline ISG15. Conserved residues and secondary structural elements are indicated**. (B)** Multi-species ISG15 sequence identity heatmap showing the homology relationship among human, feline and other representative mammalian ISG15 proteins.

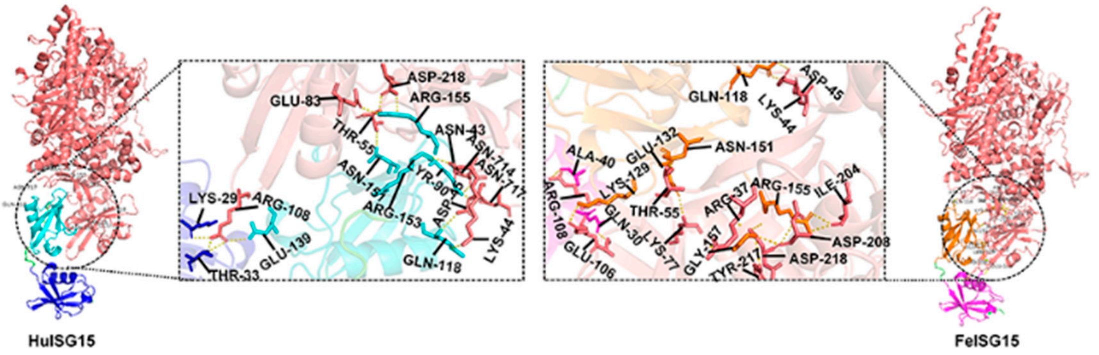

Figure S2. Predicted docking of human and feline ISG15 with FIPV protease. Predicted docking models of human and feline ISG15 with FIPV protease, with key interface residues highlighted.

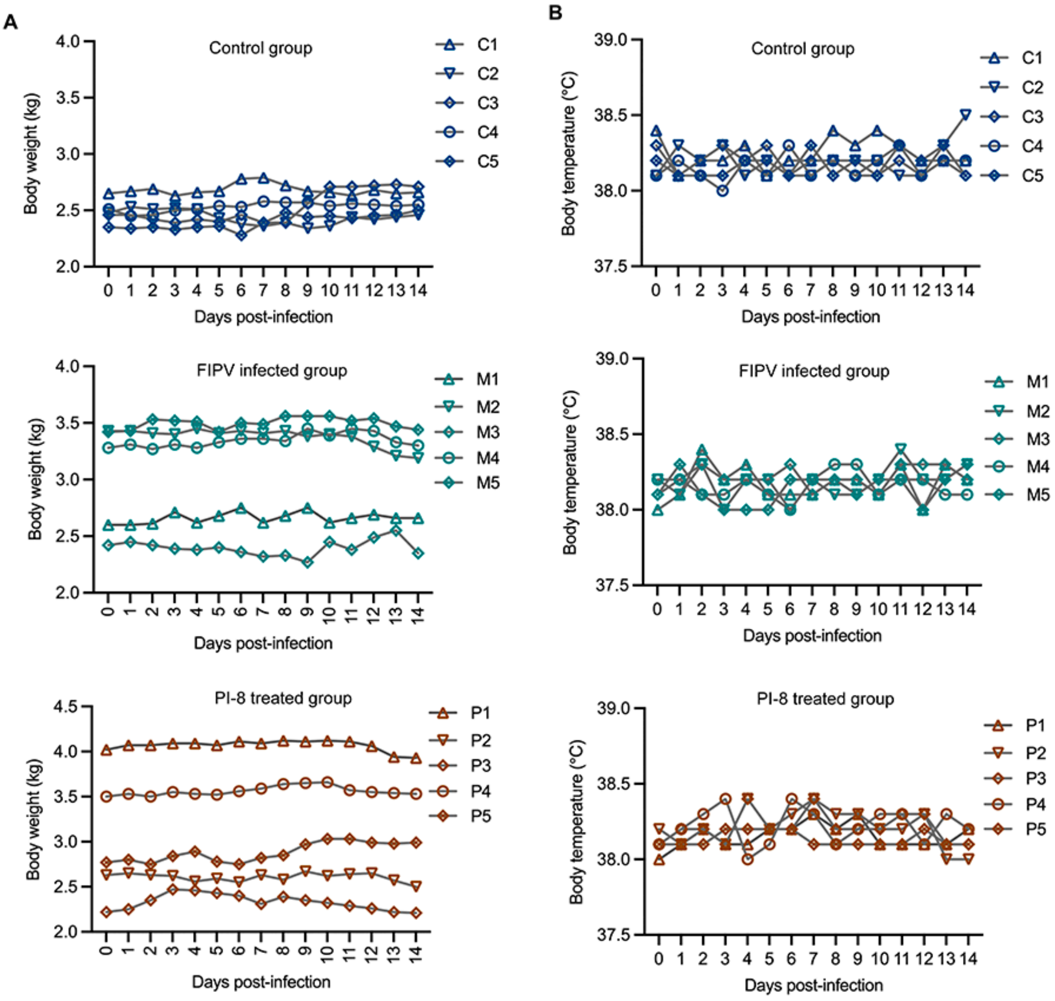

Figure S3. Body weight and body temperature of cats remain stable during FIPV infection and PI-8 treatment. **(A)** Daily body weight profiles of cats in the non-infected control, FIPV-infected model, and PI-8-treated groups during the 14-day observation period. **(B)** Daily body temperature profiles of cats in the corresponding groups. Each line represents an individual cat **(A-B).**

Table S2. Four P. americana peptides information and docking results with human ISG15.

Human	Sequence	Mass	Leading razor protein	Charges	Score	Binding Energy	Stability	Toxicity
PI-8	SKVYFQGK	955.6	Q9BPS0	2	56.1	-5.4	-21.9	0
PI-9	EGIPPDQQR	1038.5	A1E216	2	74.9	-6.4	30.7	0
PI-20	AVDTGTPVMQHYTEM-LDVIR	2275.1	H2F490	3	16.1	-5.2	19.2	0
PI-28	AAFNLPTVMENFVNQ DNIANSFSFHHGR	3176.5	A0A2P0XJ12	4	3.7	-5.6	27.32	0.1

Table S3. Four P. americana peptides information and docking results with Feline ISG15.

Feline	Sequence	Mass	Leading razor protein	Charges	Score	Binding Energy	Stability	Toxicity
PI-8	SKVYFQGK	955.6	Q9BPS0	2	56.1	-6.2	-21.9	0
PI-9	EGIPPDQQR	1038.5	A1E216	2	74.9	-7	30.7	0
PI-20	AVDTGTPVMQHYTEM-LDVIR	2275.1	H2F490	3	16.1	-5.5	19.2	0
PI-28	AAFNLPTVMENFVNQ DNIANSFSFHHGR	3176.5	A0A2P0XJ12	4	3.7	-5.4	27.32	0.1

Table S4. Information of cats.

	Cat No.	Gender	Weight (kg)	Age (Months)
FIPV infected group	M1	Female	2.60	12
	M2	Female	3.36	12
	M3	Female	2.44	13
	M4	Female	3.31	12
	M5	Female	3.43	11
PI-8 treated group	P1	Female	4.02	14
	P2	Female	2.63	11
	P3	Female	2.22	9
	P4	Female	3.50	12
	P5	Female	2.77	10
Control group	C1	Female	2.65	10
	C2	Female	2.48	11
	C3	Female	3.36	12
	C4	Female	3.10	13
	C5	Female	2.22	11

Table S5. The sequences of primers for RT-qPCR.

Primer	Sequence
FIPV-F	ATTGATGGAGTCTTCTGGGTTGC
FIPV-R	AAGCTCAAACTGTGGCGGTA
GAPDH-F	CATCAATGGAAAGCCCATCAC
GAPDH-R	CCCAGTAGACTCCACADACATAC
ISG15-F	CCGCATTCCAGCAGAGACT
ISG15-R	CCCCTCGAAGTTCAGCCAGAA
IFNA1-F	TCTCTGGGCTGTGACCTGC
IFNA1-R	GGCGAAGTCATTTCTGTCCTT
IFNB-F	GGTGCATCCTCCAAATCGCTC
IFNB-R	AGGGACCTCGAAGTTCATCCT
MX1-F	TCAGGAAACGGGACTCAAGAAG
MX1-R	GTGACAATACCGCTGCCTCTG
IL1A-F	CGAAAAGCAGGGGGAAAATACC
IL1A-R	TCTCAGGCATCTCCTTTAGCA
TNF-α-F	GAAAGCATGATCCGGGACGTG
TNF-α-R	GGGGCAGAGGGTTGATTAGTT
IL-1β-F	ACCAGTGAAATGATGGCTTACTACAG
IL-1β-R	ACTCTTGTTGTCGGGCTGG

IL-6-F	TGGGTTCAATCAGGAGACC
IL-6-R	TTCTACGGTTGGGACAGGGA
